# Perceived Monkeypox Concern and Risk among Men Who Have Sex with Men: Evidence and Perspectives from The Netherlands

**DOI:** 10.3390/tropicalmed7100293

**Published:** 2022-10-10

**Authors:** Haoyi Wang, Kennedy J. I. d’Abreu de Paulo, Thomas Gültzow, Hanne M. L. Zimmermann, Kai J. Jonas

**Affiliations:** Department of Work and Social Psychology, Maastricht University, 6200 ER Maastricht, The Netherlands

**Keywords:** monkeypox, MSM, concern about infection, perceived risk of infection, prevention

## Abstract

The current monkeypox epidemic is most prevalent among men who have sex with men (MSM). PrEP users and MSM with HIV (MSMHIV) are considered at highest risk of monkeypox infection in The Netherlands, and are being targeted for monkeypox vaccination. Together with the epidemiological evidence, perceived concern and risk are also relevant for decision making about health behaviour, e.g., vaccination uptake. It is thus timely to examine which subpopulations among MSM consider themselves to be most at risk and are most concerned about monkeypox. This study aimed to help determine if the current measures to curb the epidemic are successfully targeted or not in The Netherlands. We conducted an online survey among 394 MSM living in The Netherlands. We first calculated the prevalence and standardised prevalence ratio (SPR) of high perceived monkeypox concern/risk by PrEP-use and HIV status. We then conducted two multivariable logistic regression analyses to investigate perceived monkeypox concern/risk and their potential socio-demographic/behavioural/health/psycho-social determinants. Among the included MSM, 52% showed high perceived concern about and 30% showed high perceived risk of monkeypox infection. PrEP users (SPR = 0.83) showed a significantly lower chance of perceived concern; in addition, MSMHIV (SPR = 2.09) were found to have a significantly higher chance of perceiving high risk of monkeypox infection. In the multivariable logistic analyses, non-PrEP users (aOR = 2.55) were more likely to perceive higher concern, while MSM who were retired (aOR = 0.23) and who had had chemsex recently (aOR = 0.63) were less likely to perceive higher concern. MSMHIV (aOR = 4.29) and MSM who had an unknown/undisclosed HIV status (aOR = 6.07), who had attended private sex parties (aOR = 2.10), and who knew people who have/had monkeypox (aOR = 2.10) were more likely to perceive a higher risk for monkeypox infection. We found that high perceived risk (aOR = 2.97) and high perceived concern (aOR = 3.13) were correlated with each other. In sum, only one-third of MSM living in The Netherlands considered themselves at high risk of monkeypox infection, and only half of them reported high concern. We identified a potential discrepancy between “actual risk” and perceived risk of and concern about monkeypox among MSM in this early stage of the monkeypox epidemic in The Netherlands, especially among PrEP users and MSMHIV. More refined public health communication strategies may be needed to improve the understanding and knowledge of the “actual risk” of monkeypox infections among MSM sub-populations, to facilitate health behaviour uptake.

## 1. Introduction

Monkeypox is a zoonotic disease, described in the literature as a less-lethal relative of smallpox disease [1,2], which is caused by the orthopoxvirus. On average, a few thousand cases occur in Africa on a yearly basis. However, the rapid development of its spreading outside the core area has put scientists and the public on high alert for monkeypox [2] and led to the declaration of a public health emergency by the World Health Organization (WHO) in July 2022 [3].

In The Netherlands, 1219 cases were reported (assessed on 29 September 2022) with the majority occurring in Amsterdam [4]. Currently, the major infection routes are skin-to-skin contact and sexual contact, facilitated by frequently changing partners [2,5]. Most of the recent infections involved men who have sex with men (MSM) [4,6]. MSM are therefore considered the population at highest risk of monkeypox infection [1]. Furthermore, the current monkeypox epidemic is quite likely to be stigmatised as another “gay epidemic”, similar to the beginning of the human immunodeficiency virus (HIV) epidemic [5,7]. This notion is being corroborated in The Netherlands but also globally, due to some MSM sub-populations, such as MSM who use HIV pre-exposure prophylaxis (PrEP), and MSM with a diagnosed HIV being labelled as the most at-risk population for monkeypox [8,9]. There is also evidence that coinfection of monkeypox and HIV is possible [10]. Together with this epidemiological perspective, it is relevant to understand how members of affected communities perceive themselves in terms of monkeypox risk, and to examine which subpopulation among MSM considers themselves at most risk and is most concerned about monkeypox. Such knowledge can complement the epidemiological as well as the healthcare provider perspective and can help to determine if the current measures to curb the epidemic are sufficiently targeted or not. To close this gap, we investigated concern about and risk of monkeypox infection among MSM in The Netherlands during the onset of the epidemic.

In psychosocial theories that are used to explain preventive behaviours towards infectious diseases, beliefs regarding risks and concerns are often linked to the engagement in such preventive behaviours. For example, in the Health Belief Model [11], perceived severity and perceived risk are two of the key determinants underlying the uptake of health behaviour, and have been associated with vaccination uptake, such as HPV and COVID-19 in previous studies [12,13,14]. However, for MSM, monkeypox is a novel health risk [15,16], and individuals cannot easily fall back on previous knowledge to determine their infection risk or to gauge how concerned they should be, as they can for other infections that have a longer history in this population [17,18]. That said, both feelings of risk and concern might potentially determine MSM’s motivations to engage in protective behaviours, such as vaccination or risk reduction behaviours (e.g., reducing one’s number of sex partners)—both have been recommended for MSM in the case of monkeypox [16,19]. Two relevant scenarios are possible: there could be (1) MSM who perceive themselves to be at risk of monkeypox who are actually at risk and should therefore be concerned; and (2) MSM who do not perceive themselves to be at risk, while they are actually at risk, and thus pay less attention to the topic, which may lead to more infections [20]. Due to a lack of knowledge in regard to MSM’s feelings of risk and concern, public health and health communication interventions may not be efficiently targeted, which might make it more likely that the target group misses opportunities for monkeypox prevention. In the long run, this may result in protective interventions not being used by MSM sub-populations that are at highest risk. Thus, it is important to understand how MSM understand this epidemic and perceive themselves in terms of concerns and risks in relation to monkeypox, especially when they are considered to be the most at-risk population by healthcare authorities. Consequently, more appropriate and efficient public health interventions can be designed and implemented, to prevent transmissions among MSM.

In addition to unravelling the epidemiologic profile of the perceived monkeypox concern and risk among MSM, it is also important to identify which MSM sub-population is more likely to perceive high levels of concern and risk of monkeypox, together with its sociodemographic, behavioural, health, and psycho-social profiles, to understand whether there is a match between perceived concern and risk and actual risk determinants. A previous study has provided insights into the determinants of monkeypox vaccination and self-isolation intention among MSM in The Netherlands [16]. Even though perceived concern and risk were associated with vaccination intention [16], it is not appropriate to assume that the reported determinants also play similar roles in terms of the perceived concern and risk for monkeypox, given the different natures of these endpoints.

Therefore, this study aimed to investigate the perceived monkeypox concern and risk among MSM, and to investigate determinants of the perceived concern and risk for monkeypox in MSM in The Netherlands, to better understand the appraisal of the monkeypox epidemic among this population at the early stages of the current monkeypox epidemic.

## 2. Materials and Methods

### 2.1. Study Design and Participants

This study has a cross-sectional design. We conducted an online survey among a convenience sample consisting of 394 MSM in July 2022, of which 257 were recruited from a cohort established in 2017 [21] and 137 MSM from an online gay dating app. In this study, we only included data from respondents who indicated they were living in The Netherlands. This study was assessed and approved by the Ethics Review Committee Psychology and Neuroscience of Maastricht University (ref.188_11_02_2018_S32). Informed consent was provided by all participants. For more information on the design of the online survey, please see our previous study, which used the same data for other endpoints [16].

### 2.2. Measures

#### 2.2.1. Outcome Measures

All measures were self-reported. Participants were asked (1) “How worried are you to catch monkeypox yourself” (hereinafter perceived monkeypox concern), and (2) “How likely is it that you will catch monkeypox yourself” (hereinafter perceived monkeypox risk). These two endpoints were measured using a 1–5 Likert scale (with 1 = “Very low” and 5 = “Very high”).

Following our previous study on monkeypox vaccination and self-isolation intention [16], we also assessed sociodemographic, behavioural/health, and psychosocial determinants.

#### 2.2.2. Socio-Demographic Determinant Measures

For sociodemographic determinants, age was dichotomised as “younger than 45 years old” and “older than 45 years old”, given the median age group in our participants was 45–55 years old (for more information, see our previous study [16]). Relationship status was categorised into “Single”, “Single but dating”, “In a monogamous relationship”, and “In an open/polygamous relationship”. Education was categorised into “Lower than Bachelor”, “Bachelor”, “Master”, and “PhD or higher”. Employment status was categorised into “Employed”, “Unemployed or receiving social welfare”, “Retired”, and “Student”. Migration status was categorised into “No migration status”, “First-generation immigrant”, and “Second-generation immigrant”. For place of residence, given the fact that most of the current monkeypox cases were diagnosed in Amsterdam and the surrounding regions, we categorised this variable into “The main urban area of The Netherlands” and “The rest of the country”. The main urban area of The Netherlands includes the agglomeration of cities in the west of The Netherlands, in particular Amsterdam, Utrecht, Leiden, The Hague, and Rotterdam (in Dutch: Randstad). As the economic and political centre of The Netherlands, the Randstad accounts for approximately 50% of the national population [22].

#### 2.2.3. Behavioural/Health Determinant Measures

For behavioural/health determinants, the number of sexual partners in the previous six months were categorised into “None”, “1”, “2 to 6”, “7 to 15”, and “More than 15”. HIV status was measured based on the HIV diagnosis of the participants, and categorised into “Negative”, “Having a positive diagnosis”, and “Unknown or not disclosed”. PrEP-use status was categorised into “current PrEP users” and “PrEP-naïve or PrEP-discontinued”, which indicates a non-PrEP-using status.

We also measured past behaviours in the previous 6 months which may be associated with a higher risk of monkeypox infection, such as substance-use status and gay subculture/sexual activities [23]. For substances use, we measured whether participants had ever used any type of substance in the previous 6 months (“Ever”/“Never”); ever used any recreational drug, such as THC, MDMA, ecstasy in the previous 6 months (“Ever”/“Never”); ever had chemsex, such as using crystal meth/tina, GHB, ketamine in the previous 6 months (“Ever”/“Never”); ever used poppers in the previous 6 months (“Ever”/“Never”); ever used erectile dysfunction drugs, such as Viagra or Kamagra, in the previous 6 months (“Ever”/“Never”); or ever used alcohol in the previous 6 months (“Ever”/“Never”). For gay subculture/sexual activities, we measured whether the participants had ever visited a gay sauna in the previous 6 months (“Ever”/“Never”); ever visited a darkroom in the previous 6 months (“Ever”/“Never”); ever visited a circuit party in the previous 6 months (“Ever”/“Never”); ever visited a Pride event in the previous 6 months (“Ever”/“Never”); ever visited a gay dance club in the previous 6 months (“Ever”/“Never”); ever attended a private sex party in the previous 6 months (“Ever”/“Never”); or ever visited a fetish event in the previous 6 months (“Ever”/“Never”).

#### 2.2.4. Psycho-Social Determinant Measures

For psycho-social determinants, we measured whether the participants knew anybody who has/had monkeypox (“Yes”/“No”). We also measured their perceived problematic consequences of monkeypox: “how problematic is it to catch monkeypox” using a 1–5 Likert scale (with 1 = “Not problematic at all” and 5 = “Very problematic”). To investigate the association and potential correlation between the two endpoints, we included perceived monkeypox risk and concern for the endpoint of perceived monkeypox concern and the endpoint of perceived monkeypox risk using the 1–5 Likert scale (with 1 = “Very low” and 5 = “Very high”).

### 2.3. Statistical Analyses

**Figure 1:**
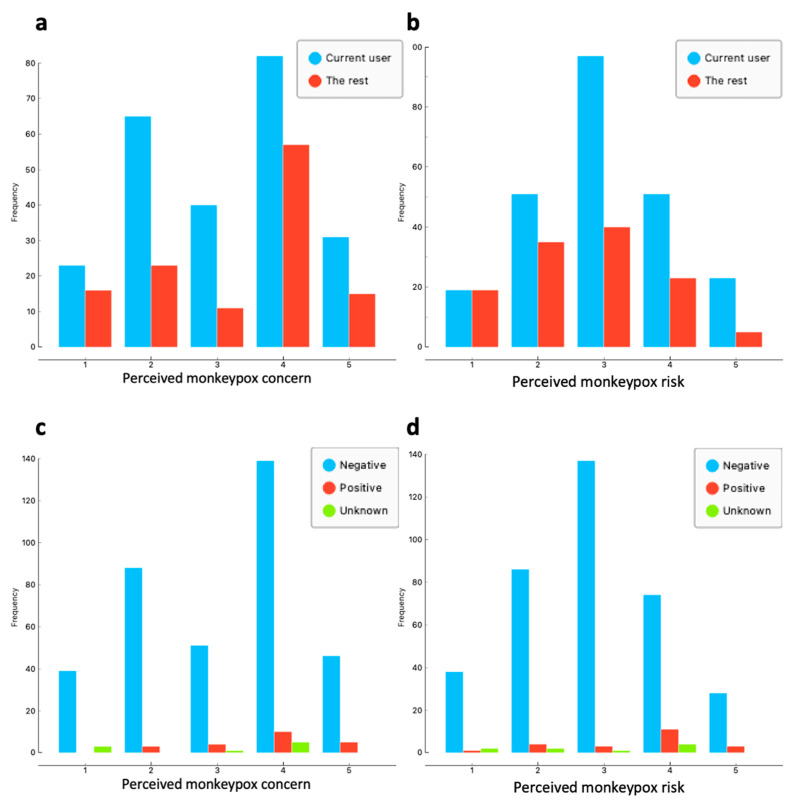
Distribution of (**a**) perceived monkeypox concern by PrEP-use status, (**b**) perceived monkeypox risk among MSM by PrEP-use status, (**c**) perceived monkeypox concern by HIV status and (**d**) perceived monkeypox risk among MSM by HIV status. Note: two endpoints were measured on a 1–5 Likert scale (with 1 = “Very low” and 5 = “Very high”).

Given the relatively small proportion of the participants showing both very high perceived monkeypox concern and risk in this study (for more details see the Results and Figure 1), and to better inform public health measures, following the analysis strategy from our previous studies [16,19] we dichotomised the two outcome endpoints as “High/very high (scale 4 and 5)” and “The rest of the scale points (scale 1–3)” for the modelling analyses.

#### 2.3.1. Descriptive Analysis

We first estimated and compared the crude prevalence and standardised prevalence ratio (SPR) of the perceived monkeypox concern and risk by PrEP-use status and HIV status, given that MSM who use PrEP and who are living with HIV are the current monkeypox vaccination priority populations in The Netherlands [8]. SPR allows the comparison of the risk levels in different sub-populations if one sub-population has a higher (SPR > 1), equal (SPR = 1) or lower (SPR < 1) probability than the overall prevalence in the total study population [22].

#### 2.3.2. Multivariable Logistic Regression Modelling

We then conducted two multivariable logistic regressions with sociodemographic, behavioural, health, and psychosocial determinants for each endpoint. Potential collinearity was not found; for the analysis, see [16]. Firstly, we conducted a univariable logistic regression with each included determinant. All determinants with a *p* < 0.10 identified in the univariable modelling analyses were retained in the multivariable model, given the relatively small sample size. All determinants with a *p* < 0.05 in the multivariable model were considered statistically significant. All analyses were conducted in R (version R 4.2.1) (R Foundation for Statistical Computing, Vienna, Austria).

## 3. Results

### 3.1. Study Population Characteristics

Of the included 394 MSM, 43% were below the age of 45, 61% were living in the Randstad of The Netherlands, 6% were living with HIV, 66% were currently using PrEP, 26% had had chemsex, 40% had attended private sex parties in the previous six months, and 17% knew people who have/had monkeypox (see Table 1 for other study population characteristics).

### 3.2. Perceived Monkeypox Concern and Risk among MSM

Among the included MSM, 39% showed “high” and 13% “very high” perceived monkeypox concern, and 23% showed “high” and 8% “very high” perceived monkeypox risk. Figure 1 summarises the frequencies of the observation of (a) perceived monkeypox concern by PrEP-use status, (b) perceived monkeypox risk by PrEP-use status, (c) perceived monkeypox concern by HIV status, (d) perceived monkeypox risk by HIV status.

After combining the “High” and “Very high” scales for the endpoints, among the total sample 52% showed high/very high perceived monkeypox concern, and 30% showed high/very high perceived monkeypox risk. When comparing results by PrEP-use status, only current PrEP users (prevalence = 47%, SPR = 0.83) showed a significantly lower probability of perceived monkeypox concern compared with non-PrEP users. No significant difference of the probability of high/very high perceived monkeypox risk was found between current PrEP users (prevalence = 31%, SPR = 1.09) and non-PrEP users (prevalence = 23%, SPR = 0.82). When comparing results by HIV status, no significant difference of the probability of high/very high perceived monkeypox concern was found between MSM living without HIV (prevalence = 51%, SPR = 0.98), MSM living with HIV (prevalence = 68%, SPR = 1.31), and MSM whose HIV status was unknown or not disclosed (prevalence = 56%, SPR = 1.06). Only MSM living with HIV (prevalence = 64%, SPR = 2.09) were found to have a significantly higher probability of perceived high/very high monkeypox risk, compared with MSM with other HIV status. See Table 2 for more information of the estimated prevalence and SPR of perceived concern and risk by PrEP-use status and HIV status.

### 3.3. Determinants of Perceived Concern and Risk among MSM

After conducting the univariable logistic regression models for both endpoints, following our statistical analysis strategy, age, relationship status, education, employment, place of residence, PrEP-use status, recreational drugs use, chemsex, erectile dysfunction medication use in the previous 6 months, knowing anybody who has/had monkeypox, and perceived risk of monkeypox infection (measured using 1–5 Likert scales) were included in the multivariable logistic regression model for the perceived monkeypox concern endpoint. Place of residence, HIV status, chemsex, erectile dysfunction medication use in the previous 6 months, having visited (1) a gay sauna, (2) a darkroom, (3) a circuit party and (4) private sex parties in the previous 6 months, knowing anybody who has/had monkeypox, and perceived concern about monkeypox infection (measured using 1–5 Likert scales) were included in the multivariable logistic regression model for the perceived monkeypox risk endpoint.

For perceived concern, no socio-demographic determinants were found to be statistically associated with the endpoint. Among behavioural/health determinants, MSM who did not use PrEP (adjusted odds ratio (aOR) = 2.55) were more likely to show high/very high levels of concern about a possible monkeypox infection. On the other hand, MSM who had had chemsex in the previous six months (aOR = 0.44) were less likely to show a high/very high concern about a possible monkeypox infection. Among psycho-social determinants, MSM who had a higher perceived risk of monkeypox (aOR = 3.26) were more likely to perceive more concern for becoming infected with monkeypox.

For perceived risk, similarly, no sociodemographic determinants were found to be associated with this endpoint. Among behavioural and health determinants, MSM who had a diagnosed HIV status (aOR = 4.29) and an unknown HIV status (aOR = 6.07), and who had ever attended private sex parties in the previous 6 months (aOR = 2.10), perceived themselves at higher risk of monkeypox. PrEP-use status was not associated with perceived risk univariably or multivariably. Among psychosocial determinants, MSM who knew anybody who has/had monkeypox (aOR = 2.60) and who perceived a higher monkeypox concern (aOR = 3.24), perceived themselves at a higher risk of monkeypox. For results obtained from univariable logistic regression analyses for both endpoints, see Table 3.

## 4. Discussion

To our knowledge, this study is the first study to report perceived concern about and risk of monkeypox and their determinants among MSM. Our findings, based on 394 MSM in The Netherlands showed that 52% of our respondents showed high/very high levels of perceived concern about monkeypox, but only 30% perceived themselves to be at high/very high risk of monkeypox, based on data collected at an early stage of the monkeypox epidemic, prior to vaccination implementation. In addition, these results highlight the fact that there may be a discrepancy between the “actual risk” according to an epidemiolocal viewpoint [1,5,15,24,25] and the perceived risk and concern among MSM living in The Netherlands. Thus, these results indicate a lack of understanding and knowledge of the ongoing monkeypox epidemic among MSM in The Netherlands.

Not surprisingly, some of the included psychosocial determinants play a role for both endpoints in our analyses, such as knowing people with monkeypox. Proximity to the health threat is a typical determinant determining concern and risk perception [26]. Our results also indicated that perceived concern about monkeypox and perceived risk of monkeypox among MSM were positively associated with each other, in a similar way to perceived severity and perceived susceptibility in the Health Belief Model [11], which could indicate that beliefs in relation to concerns and risks about monkeypox should be targeted simultaneously. Another potential explanation for this finding could be that the two endpoints do in fact reflect an underlying psychological construct, such as perceived threat in the Health Belief Model. However, we also identified other determinants which may influence both determinants among MSM differently.

While non-PrEP users and current PrEP users showed similar levels of perceived risk of monkeypox, non-PrEP users showed a significantly higher concern about being infected with monkeypox, compared with current PrEP users. This may indicate a potential interaction effect from the current monkeypox vaccination strategy which is predominantly focusing on PrEP-using MSM [8]. As non-PrEP users cannot access the monkeypox vaccine at the moment in The Netherlands, they may consider themselves not protected against the monkeypox epidemic, which may thus lead to a higher perceived concern about monkeypox infection even when sharing similar risk beliefs as current PrEP users. Another reason may be the lack of knowledge and understanding of monkeypox among MSM, especially among the current PrEP users. After being risk-free of acquiring HIV by using PrEP [27], PrEP-users may have a lower perception of monkeypox risk, and at the same time perceive a monkeypox infection as less severe than HIV. This finding dovetails nicely with other results showing that severity perceptions regarding STIs are also lower while using PrEP [18,27]. Given the significantly lower perceived concern about monkeypox infection among PrEP users, public health communication may be needed to improve the current understanding of monkeypox as a novel health risk which cannot be covered by PrEP. However, before this, further research should explore the underlying reasons for the differences in perceived risks.

Similarly, but in an opposite direction, HIV status also played differential roles in the level of perceived monkeypox concern and risk among MSM. A positive and unknown/non-disclosed HIV status predicted a higher likelihood of perceived risk but not a higher perceived concern about monkeypox, among MSM. This finding could reflect an awareness of risky sexual behaviour and a higher risk of monkeypox infection that is perceived among this sub-population of MSM. However, given their experience of HIV infection risk, they may not regard an infection with monkeypox to be as problematic as an HIV infection. This was similar to the perceptions of being infected with other STIs, among people living with HIV [28,29]. In addition, another reason for this finding may be the unknown risk and pathways of developing more severe disease outcomes among people living with HIV [25]. Even though a higher risk of monkeypox infection is perceived among this population, it is still currently unknown whether an HIV infection alters a person’s risk of acquiring monkeypox after exposure [25], and MSM with HIV or unknown HIV status may not develop a higher perceived monkeypox concern for this infection.

While our previous study reported no behavioural determinants associated with monkeypox vaccination intention among MSM in The Netherlands [16], we identified several past behaviours associated with perceived monkeypox concern and perceived monkeypox risk among MSM, both univariably and multivariably, indicating that past behaviours are associated with vaccination intentions and perceived monkeypox concern and perceived monkeypox risk differently. For example, our study showed that people who had had chemsex in the previous six months showed a lower likelihood of perceiving high/very high monkeypox concern. One possible reason may be that MSM who engage in chemsex tend to underestimate their risk for infections that can be transmitted via sexual contact in general [30]. Our study suggests a potential similar mechanism behind the lower perceived risk of monkeypox among MSM who had had chemsex. In addition, based on the multivariable model, not surprisingly, MSM who had attended private sex parties in the previous six months were more likely to perceive themselves as having high/very high risk for monkeypox. An elevated number of sexual partners and sexual activities may be associated with this behaviour (a potential negative association was shown between a higher number of sexual partners and never attending a private party, shown in our collinearity analysis [16]), thus leading to a higher perceived risk of monkeypox, which indicates that this sub-population of MSM indeed perceive a higher risk when they are actually at risk for monkeypox. Therefore, there is at least some evidence that certain sub-populations gauge their risk correctly.

Although MSMs’ place of residence was found to be associated with both perceived monkeypox concern and perceived monkeypox risk univariably, such effects disappeared after adjusting for other determinants. Given the fact that most of monkeypox cases were reported from Amsterdam [4], we hypothesised a higher perceived monkeypox concern and perceived monkeypox risk among MSM from the Randstad. However, no differences in both endpoints between Randstad and the rest of the country were found. One reason may be due to the ecological fallacy. Even though Amsterdam rests within the Randstad region, the internal heterogeneity of the likelihood of perceived monkeypox concern and perceived monkeypox risk between different cities within Randstad can be masked when aggregating data on the Randstad level. It could also be that the MSM from the Randstad differ regarding the other included variables compared with MSM from other regions, which led to the effects disappearing in the multivariable models.

### Limitations and Recommendations for Future Research

Despite the novelty and timely communication of this study on perceived monkeypox concern and risk among MSM with evidence and perspectives from The Netherlands, there are some limitations that are applicable to our study.

Firstly, we suggested a potential interaction between vaccination strategy and PrEP-use status. However, due to insufficient data, future studies are warranted and should further investigate such an interaction with both more qualitative and empirical evidence. Secondly, our data were collected at the beginning stage of the monkeypox epidemic, while comprehensive public health communication and public health measures (i.e., vaccination) were just starting up. This may have influenced MSM’s current knowledge of monkeypox, and, in turn, have led to underestimated levels of perceived concern and risk about monkeypox among MSM. However, as measuring knowledge fell outside the scope of this research, we cannot be sure if this is the case. An updated assessment at a later stage could be warranted, to measure the change in perceived concern and risk of monkeypox, since more information has been shared with the public in the meantime. Nevertheless, future case development can be taken into account. Thirdly, we did not measure the potential aversive attitudes towards governmental prevention measures that are associated with a restriction of personal freedom. As a consequence, our findings may be an underestimation. An updated assessment should therefore also include this assessment, for a more comprehensive understanding of the perceived concern and risk about monkeypox among MSM in The Netherlands. Fourthly, given the relatively small sample size in this study, the power of our models may be limited. For example, our models did not have the power to investigate the covariations from the geo-location on a finer geographic scale such as public health services at a regional level (25 in total), or municipality level (345 in total). Our results on the spatial perspective may thus be limited and not comprehensive enough to support local monkeypox prevention. Therefore, future studies could zoom in on a more refined geographical level, to provide more concise estimations with a larger sample size. Lastly, given that our data were self-reported, our data were thus not devoid of information biases, especially on past risky behaviours. This may result in biased parameters.

## 5. Conclusions

In conclusion, only a small proportion of MSM living in The Netherlands considered themselves to be at a high/very high risk of monkeypox infection, and around half of them showed a high/very high concern about it. We found that the current PrEP users and non-PrEP users shared a similar perceived monkeypox risk of monkeypox infection, but non-PrEP users were more concerned about monkeypox infections. A potential discrepancy between the “actual risk” and the perceived risk and concern about monkeypox may exist among MSM in this early stage of the monkeypox epidemic. Public health professionals should therefore put more effort into improving understanding and knowledge of the “actual risk” of monkeypox infections among these MSM sub-populations, to facilitate health behaviour uptake by means of improved public health and health communication interventions.

## Figures and Tables

**Table 1:** Study characteristics.

Variables		Total Sample (n = 394)		
			N	%
Socio-demographic determinants	Age			
		<45 years	171	43.4
		>45 years	223	56.6
	Relationship			
		Single	79	20.05
		Single but dating	91	23.1
		Monogamous relationship	35	8.88
		Open/Polyamorous relationship	189	47.97
	Education			
		Lower than Bachelor	89	22.65
		Bachelor	131	33.33
		Master	142	36.13
		PhD or higher	31	7.89
	Employment			
		Employed	335	85.03
		Unemployed or receiving social welfare	22	5.58
		Retired	20	5.08
		Student	17	4.31
	Migration status			
		No migration status	325	82.91
		First-generation migrant	51	13.01
		Second-generation migrant	16	4.08
	Residence			
		The rest of the country	154	39.10
		Randstad (main urban area)	240	60.90
Behavioural and Health determinants	Number of sexual partners in the previous 6 months			
		None	8	2.03
		1	46	11.68
		2 to 6	82	20.81
		7 to 15	159	40.36
		More than 15	99	25.13
	HIV status			
		HIV-negative	363	92.13
		HIV-positive	22	5.58
		HIV status unknown or not disclosed	9	2.28
	PrEP-use status			
		Current PrEP users	241	66.39
		PrEP-naïve or PrEP-discontinued	122	30.96
	Any type of substance use in the previous 6 months			
		Never	45	11.42
		Ever	349	88.58
	Recreational drugs use in the previous 6 months ^1^			
		Never	250	63.45
		Ever	144	36.55
	Chemsex in the previous 6 months ^2^			
		Never	293	74.37
		Ever	101	25.63
	Poppers use in the previous 6 months			
		Never	183	46.45
		Ever	211	53.55
	Erectile dysfunction medication use in the previous 6 months ^3^			
		Never	228	57.87
		Ever	166	42.13
	Alcohol use in the previous 6 months			
		Never	93	23.6
		Ever	301	76.4
	Visited a gay sauna in the previous 6 months			
		Never	251	63.71
		Ever	143	36.29
	Visited a darkroom in the previous 6 months			
		Never	238	60.41
		Ever	156	39.59
	Visited a circuit party in the previous 6 months			
		Never	283	71.83
		Ever	111	28.17
	Visited a Pride event in the previous 6 months			
		Never	203	51.52
		Ever	191	48.48
	Visited a gay dance club in the previous 6 months			
		Never	137	34.77
		Ever	257	65.23
	Attended private sex parties in the previous 6 months			
		Never	277	70.3
		Ever	117	29.7
	Visited fetish events/fairs in the previous 6 months			
		Never	308	78.17
		Ever	86	21.83
Psycho-social determinants	Knowing anybody who has/had monkeypox			
		No	326	82.74
		Yes	68	17.26
	Perceived problematic consequences of monkeypox *		4	[3,4]

Notes: ^1^ I use substances recreationally (for example THC, MDMA, ecstasy, etc). ^2^ I use substances in the context of sex (for example crystal meth/tina, GHB, ketamine etc.). ^3^ I use erectile dysfunction medication (for example Viagra, Kamagra). * Indicates variable with a 1–5 Likert scale, with 1 = extremely unlikely and 5 = extremely likely), results were reported in median [interquartile range].

**Table 2:** Prevalence and standardised prevalence ratio of perceived monkeypox concern and risk among MSM in The Netherlands, July 2022.

Sub-Population	Perceived Monkeypox Concern (High/Very High vs. Rest of Scale) *					Perceived Monkeypox Risk (High/Very High vs. Rest of Scale) *				
	n	Prevalence (%)	95%CI	SPR	95%CI	n	Prevalence (%)	95%CI	SPR	95%CI
Total sample (N = 394)	205	52.03	47.10–56.92	NA	NA	120	30.46	26.12–35.17	NA	NA
PrEP users (N = 241)	113	46.89	40.69–53.19	0.83	0.68–0.99	74	30.71	25.22–36.79	1.09	0.86–1.36
Non-PrEP users (N = 122)	72	59.02	50.14–67.34	1.05	0.82–1.30	28	22.95	16.38–21.16	0.82	0.54–1.15
HIV positive (N = 22)	15	68.18	47.31–83.63	1.31	0.73–2.05	14	63.64	42.95–80.27	2.09	1.14–3.32
HIV negative (N = 363)	185	50.96	45.84–56.07	0.98	0.84–1.13	102	28.10	23.72–32.93	0.92	0.75–1.11
HIV status unknown/undisclosed (N = 9)	5	55.56	26.67–81.12	1.06	0.34–2.21	4	44.44	18.88–73.33	1.45	0.38–3.23

Note: CI: confidence interval; NA: not applicable; PrEP: pre-exposure prophylaxis; SPR: standardised prevalence ratio. * 1–5 Likert scale, with 1 = extremely unlikely and 5 = extremely likely).

**Table 3:** Determinants of perceived concern about and perceived risk of getting monkeypox among MSM in The Netherlands, July 2022.

Variables			Perceived Monkeypox Concern (High and Very High vs. Rest of Scale) **						Perceived Monkeypox Risk (High and Very High vs. Rest of Scale) **					
			Univariable Model			Multivariable Model			Univariable Model			Multivariable Model		
			OR	95%CI	*p*-Value	aOR	95%CI	*p*-Value	OR	95%CI	*p*-Value	aOR	95%CI	*p*-Value
Socio-demographic determinants	Age													
		<45 years	ref.	–	–	ref.	–	–	ref.	–	–			
		>45 years	0.53	0.36–0.79	0.002	0.93	0.54–1.62	0.806	0.91	059–1.40	0.672			
	Relationship													
		Single	ref.	–	–				ref.	–	–			
		Single but dating	1.25	0.68–2.29	0.486				0.99	0.50–1.96	0.975			
		Monogamous relationship	0.86	0.39–1.92	0.719				0.69	0.26–1.81	0.453			
		Open/Polyamorous relationship	1.15	0.68–1.95	0.597				1.55	0.87–2.77	0.138			
	Education													
		Lower than Bachelor	ref.	–	–	ref.	–	–	ref.	–	–			
		Bachelor	1.41	0.82–2.42	0.219	1.37	0.67–2.78	0.387	0.88	0.49–1.57	0.658			
		Master	1.68	0.99–2.87	0.056	1.81	0.89–3.68	0.103	0.81	0.46–1.44	0.476			
		PhD or higher	2.44	1.05–5.69	0.039	1.73	0.60–4.98	0.309	1.31	0.56–3.05	0.536			
	Employment													
		Employed	ref.	–	–	ref.	–	–	ref.	–	–			
		Unemployed or receiving social welfare	0.61	0.23–1.64	0.328	0.83	0.23–3.03	0.775	0.46	0.13–1.64	0.234			
		Retired	0.26	0.09–0.71	0.009	0.23	0.06–0.82	0.002	0.64	0.23–1.77	0.385			
		Student	2.04	0.76–5.41	0.156	1.73	0.54–5.49	0.351	0.93	0.35–2.48	0.878			
	Migration status													
		No migration status	ref.	–	–				ref.	–	–			
		First-generation migrant	1.14	0.63–2.05	0.670				0.94	0.49–1.79	0.845			
		Second-generation migrant	0.73	0.26–2.00	0.536				0.75	0.24–2.38	0.626			
	Place of residence													
		The rest of the Country	ref.	–	–	ref.	–	–	ref.	–	–	ref.	–	–
		Randstad (main urban area)	1.92	1.27–2.89	0.002	1.47	0.87–2.49	0.155	1.67	1.06–2.64	0.027	1.30	0.74–2.29	0.360
Behavioural and health determinants	Number of sexual partners in the previous 6 months													
		None	ref.	–	–				ref.	–	–			
		1	1.40	0.30–2.51	0.669				0.30	0.06–1.55	0.150			
		2 to 6	1.77	0.41–7.67	0.443				0.60	0.14–2.61	0.495			
		7 to 15	2.00	0.45–8.83	0.360				0.83	0.19–3.70	0.811			
		More than 15	2.03	0.45–9.05	0.355				1.24	0.27–5.54	0.777			
	HIV status													
		HIV negative	ref.	–	–				ref.	–	–	ref.	–	–
		HIV positive	2.06	0.82–5.18	0.123				4.48	1.82–11.00	0.001	4.29	1.44–12.82	0.009
		HIV status unknown or not disclosed	1.20	0.31–4.55	0.786				2.04	0.53–7.78	0.292	6.07	1.24–29.79	0.026
	PrEP-use status													
		Current PrEP users	ref.	–	–	ref.	–	–	ref.	–	–			
		PrEP-naïve or PrEP-discontinued	1.63	1.04–2.53	0.030	2.55	1.39–4.67	0.002	0.67	0.41–1.11	0.122			
	Any type of substance use in the previous 6 months													
		Never	ref.	–	–				ref.	–	–			
		Ever	1.60	0.85–3.02	0.149				1.16	0.60–2.25	0.565			
	Recreational drugs use in the previous 6 months ^1^													
		Never	ref.	–	–	ref.	–	–	ref.	–	–			
		Ever	1.56	1.03–2.36	0.035	1.50	0.86–2.62	0.156	1.44	0.92–2.33	0.105			
	Chemsex in the previous 6 months ^2^													
		Never	ref.	–	–	ref.	–	–	ref.	–	–	ref.	–	–
		Ever	0.63	0.40–0.99	0.0491	0.44	0.22–0.88	0.021	1.55	0.96–2.50	0.071	1.23	0.60–2.71	0.605
	Poppers in the previous 6 months													
		Never	ref.	–	–				ref.	–	–			
		Ever	1.01	0.68–1.50	0.965				1.39	0.90–2.14	0.140			
	Erectile dysfunction medication use													
	in the previous 6 months ^3^													
		Never	ref.	–	–	ref.	–	–	ref.	–	–	ref.	–	–
		Ever	0.70	0.47–1.05	0.088	1.16	0.63–2.15	0.625	0.98–2.32	0.47–1.05	0.062	1.25	0.67–2.34	0.479
	Alcohol use in the previous 6 months													
		Never	ref.	–	–				ref.	–	–			
		Ever	0.97	0.61–1.54	0.885				0.79	0.48–0.79	0.344			
	Visited a gay sauna in the previous 6 months													
		Never	ref.	–	–				ref.	–	–	ref.	–	–
		Ever	1.28	0.84–1.93	0.241				1.28	0.68–2.41	0.439	1.28	0.68–2.41	0.439
	Visited a darkroom in the previous 6 months													
		Never	ref.	–	–				ref.	–	–	ref.	–	–
		Ever	1.08	0.72–1.62	0.706				1.43	0.74–2.76	0.294	1.43	0.74–2.76	0.294
	Visited a circuit party in the previous 6 months													
		Never	ref.	–	–				ref.	–	–	ref.	–	–
		Ever	1.06	0.69–1.65	0.780				0.89	0.44–0.81	0.749	0.89	0.44–0.81	0.749
	Visited a Pride event in the previous 6 months													
		Never	ref.	–	–									
		Ever	1.11	0.75–1.65	0.597									
	Visited a gay dance club in the previous 6 months													
		Never	ref.	–	–									
		Ever	1.16	0.76–1.75	0.487									
	Attended private sex parties in the previous 6 months													
		Never	ref.	–	–				ref.	–	–	ref.	–	–
		Ever	0.87	0.56–1.34	0.526				2.10	1.04–4.24	0.037	2.10	1.04–4.24	0.037
	Visited fetish events/fairs in the previous 6 months													
		Never	ref.	–	–				ref.	–	–			
		Ever	1.08	0.67–1.74	0.760				1.39	0.84–2.29	0.204			
Psycho-social determinants	Knowing anybody who has/had monkeypox													
		No	ref.	–	–	ref.	–	–	ref.	–	–	ref.	–	–
		Yes	2.81	1.59–4.98	<0.001	1.58	0.76–3.27	0.218	3.77	2.20–6.47	<0.001	2.60	1.33–5.07	0.005
	Perceived problematic consequences of monkeypox *		1.18	0.96-1.45	0.101				1.07	0.86–1.33	0.560			
	Perceived concern about monkeypox infection *		NA	NA	NA	NA	NA	NA	3.13	2.36–4.13	<0.001	3.24	2.40–4.38	<0.001
	Perceived risk of monkeypox infection *		2.97	2.31-3.82	<0.001	3.26	0.81–1.36	<0.001	NA	NA	NA	NA	NA	NA

Notes: ^1^ I use substances recreationally (for example THC, MDMA, ecstasy, etc). ^2^ I use substances in the context of sex (for example, crystal meth/tina, GHB, ketamine etc.). ^3^ I use erectile dysfunction medication (for example Viagra, Kamagra). * indicates a variable with a 1–5 Likert scale, with 1 = extremely unlikely and 5 = extremely likely). ** indicates a variable with a 1–5 Likert scale, with 1 = “very low” and 5 = “very high”). NA = not applicable.

## Data Availability

The data presented in this study are available on request from the corresponding author.
